# Supplementary material for: Forecasting the flooding dynamics of flatwoods salamander breeding wetlands under future climate change scenarios
Source: PeerJ. 2023 Sep 19;11:e16050. doi: 10.7717/peerj.16050 (PMC10516105; doi:10.7717/peerj.16050)
Supplement: Table S1 [file peerj-11-16050-s001.docx]

Table S1. Additional details about water level monitoring in pine flatwoods wetlands on Eglin Air Force Base, Florida.

| Wetland  ID | Start date | End date | Training points | Testing points | Distance to closest dry well (km) | Distance to closest rain gauge (km) |
| --- | --- | --- | --- | --- | --- | --- |
| 1 | 11-26-2014 | 2-7-2022 | 1512 | 505 | 0.80 | 0.16 |
| 2 | 11-26-2014 | 2-7-2022 | 1620 | 541 | 0.63 | < 0.01 |
| 3A | 11-26-2014 | 2-7-2022 | 1646 | 549 | 0.24 | 0.81 |
| 3B | 11-26-2014 | 2-6-2022 | 1743 | 581 | 0.24 | 0.81 |
| 4 | 11-26-2014 | 5-2-2022 | 1753 | 585 | 0.05 | 0.44 |
| 5 | 11-26-2014 | 5-2-2022 | 1512 | 504 | 0.41 | 0.01 |
| 7 | 11-26-2014 | 2-2-2022 | 1529 | 510 | 2.61 | 0.17 |
| 10 | 12-14-2017 | 2-2-2022 | 829 | 277 | 2.16 | 0.04 |
| 12 | 11-26-2014 | 2-7-2022 | 1528 | 510 | 2.44 | 1.83 |
| 13 | 11-26-2014 | 2-7-2022 | 1463 | 488 | 0.22 | 0.49 |
| 14 | 11-14-2015 | 2-7-2022 | 1292 | 431 | 2.32 | 1.78 |
| 15 | 11-26-2014 | 2-3-2022 | 1563 | 522 | 5.20 | 0.01 |
| 16 | 11-9-2017 | 2-3-2022 | 795 | 266 | 3.29 | 1.93 |
| 19 | 8-19-2015 | 2-3-2022 | 1560 | 521 | 3.17 | 2.02 |
| 21 | 11-14-2015 | 2-7-2022 | 1139 | 380 | 1.32 | 0.88 |
| 30A | 8-14-2015 | 2-7-2022 | 1223 | 408 | 0.90 | 0.38 |
| 30B | 11-14-2015 | 2-7-2022 | 1317 | 440 | 0.90 | 0.38 |
| 31 | 10-14-2015 | 2-7-2022 | 1203 | 401 | 0.74 | 0.20 |
| 32 | 8-19-2015 | 2-3-2022 | 1542 | 514 | 5.10 | 0.13 |
| 33 | 8-13-2015 | 2-3-2022 | 1439 | 480 | 4.82 | 0.40 |
| 34 | 8-13-2015 | 2-3-2022 | 1203 | 402 | 4.30 | 0.91 |
| 36 | 8-20-2015 | 2-7-2022 | 1239 | 413 | 1.48 | 0.91 |
| 40A | 10-14-2015 | 2-6-2022 | 1461 | 488 | 0.14 | 0.52 |
| 40B | 10-14-2015 | 2-6-2022 | 1147 | 383 | 0.14 | 0.52 |
| 41 | 8-20-2015 | 2-3-2022 | 1206 | 403 | 8.16 | 3.93 |
| 49 | 8-20-2015 | 5-2-2022 | 1382 | 461 | 0.98 | 0.68 |
| 50 | 8-19-2015 | 5-2-2022 | 1530 | 511 | 2.06 | 1.93 |
| 51 | 8-19-2015 | 5-2-2022 | 1392 | 465 | 1.66 | 1.35 |
| 52 | 12-14-2017 | 5-2-2022 | 1058 | 353 | 1.74 | 1.49 |
| 53 | 8-15-2015 | 5-2-2022 | 1615 | 539 | 0.32 | 0.31 |
| 107 | 8-15-2015 | 12-2-2021 | 904 | 302 | 2.74 | 0.28 |
| 112 | 8-19-2015 | 2-3-2022 | 1455 | 486 | 5.07 | 0.71 |
| 202 | 8-14-2015 | 2-7-2022 | 1383 | 461 | 0.44 | 0.41 |
| 212 | 9-10-2015 | 5-2-2022 | 1380 | 461 | 0.49 | 0.91 |
| 215 | 11-9-2017 | 2-3-2022 | 896 | 299 | 5.19 | 0.29 |
