# Supplementary material for: Forecasting the flooding dynamics of flatwoods salamander breeding wetlands under future climate change scenarios
Source: PeerJ. 2023 Sep 19;11:e16050. doi: 10.7717/peerj.16050 (PMC10516105; doi:10.7717/peerj.16050)
Supplement: Table S2 — Median, lower, and upper 95% highest density posterior distributions for model parameters and wetland basins. Model parameters included an intercept (α), an error term (σ), precipitation (βRAIN), an autoregression term (βAR1), total precipitation over the previous seven days (βWEEKRAIN), the 12-month standardized precipitation-evapotranspiration index (βSPEI), potential evapotranspiration (βPET), and associated interactions and quadratic effects. [file peerj-11-16050-s002.docx]

Table S2a. Median, lower, and upper 95% highest density posterior distributions for model parameters and wetland basins. Model parameters included an intercept (α), an error term (σ), precipitation (βRAIN), an autoregression term (βAR1), total precipitation over the previous seven days (βWEEKRAIN), the 12-month standardized precipitation-evapotranspiration index (βSPEI), potential evapotranspiration (βPET), and associated interactions and quadratic effects.

| ID | α | βAR1 | βRAIN | βRAIN:AR1 | βPET | βPET^2^ |
| --- | --- | --- | --- | --- | --- | --- |
| 1 | 0.78 (-0.67, 2.17) | 0.94 (0.93, 0.96) | 6.65 (5.83, 7.40) | -0.29 (-0.32, -0.26) | -0.49 (-1.17, 0.17) | -0.02 (-0.10, 0.05) |
| 2 | 1.06 (0.06, 2.06) | 0.98 (0.97, 0.98) | 3.70 (3.19, 4.22) | -0.10 (-0.12, -0.08) | -0.60 (-1.06, -0.12) | 0.01 (-0.03, 0.06) |
| 3A | -0.12 (-1.34, 1.00) | 0.97 (0.96, 0.98) | 3.03 (2.64, 3.46) | -0.06 (-0.08, -0.04) | -0.24 (-0.78, 0.33) | -0.03 (-0.09, 0.03) |
| 3B | 0.04 (-0.85, 1.02) | 0.98 (0.97, 0.98) | 2.74 (2.37, 3.13) | -0.08 (-0.09, -0.06) | -0.15 (-0.58, 0.3) | -0.01 (-0.07, 0.02) |
| 4 | -0.94 (-2.31, 0.44) | 0.98 (0.97, 0.99) | 4.84 (4.28, 5.33) | -0.25 (-0.27, -0.23) | -0.13 (-0.79, 0.46) | -0.03 (-0.10, 0.03) |
| 5 | -0.68 (-2.43, 0.98) | 0.98 (0.97, 0.99) | 3.61 (2.82, 4.33) | -0.17 (-0.19, -0.15) | -0.25 (-1.00, 0.51) | -0.03 (-0.12, 0.04) |
| 7 | -1.75 (-3.21, -0.33) | 0.98 (0.97, 0.99) | 6.61 (5.70, 7.54) | -0.15 (-0.18, -0.13) | 0.33 (-0.33, 0.97) | -0.06 (-0.13, 0.01) |
| 10 | -0.54 (-2.19, 1.25) | 0.97 (0.96, 0.98) | 3.60 (2.24, 5.07) | -0.11 (-0.15, -0.07) | -0.55 (-1.31, 0.23) | 0.00 (-0.07, 0.09) |
| 12 | 0.73 (-0.41, 1.76) | 0.98 (0.97, 0.99) | 2.63 (2.27, 3.00) | -0.06 (-0.07, -0.05) | -0.55 (-1.07, -0.02) | 0.02 (-0.04, 0.08) |
| 13 | 1.47 (0.08, 2.82) | 0.95 (0.94, 0.96) | 3.06 (2.60, 3.57) | -0.08 (-0.1, -0.06) | -0.93 (-1.59, -0.29) | 0.03 (-0.04, 0.10) |
| 14 | 1.14 (-0.13, 2.44) | 0.98 (0.97, 0.99) | 2.62 (2.10, 3.13) | -0.05 (-0.07, -0.03) | -0.70 (-1.3, -0.09) | 0.03 (-0.02, 0.1) |
| 15 | 1.02 (0.05, 1.96) | 0.97 (0.96, 0.98) | 2.90 (2.45, 3.28) | -0.07 (-0.09, -0.05) | -0.63 (-1.09, -0.21) | 0.03 (-0.01, 0.08) |
| 16 | 1.28 (-0.27, 2.81) | 0.98 (0.97, 1.00) | 1.95 (1.14, 2.83) | -0.20 (-0.25, -0.14) | -0.89 (-1.57, -0.14) | 0.05 (-0.02, 0.12) |
| 19 | 0.45 (-0.13, 1.01) | 0.99 (0.99, 1.00) | 2.58 (2.35, 2.81) | -0.06 (-0.07, -0.05) | -0.5 (-0.78, -0.22) | 0.02 (0.00, 0.05) |
| 21 | 0.39 (-1.00, 1.77) | 0.97 (0.96, 0.98) | 3.01 (2.42, 3.64) | -0.18 (-0.22, -0.14) | -0.22 (-0.89, 0.44) | -0.02 (-0.09, 0.05) |
| 30A | -0.22 (-1.22, 0.77) | 0.98 (0.97, 0.99) | 4.04 (3.47, 4.61) | -0.27 (-0.31, -0.23) | 0.00 (-0.52, 0.46) | -0.04 (-0.01, 0.00) |
| 30B | 0.10 (-0.72, 0.93) | 0.99 (0.98, 0.99) | 5.15 (4.62, 5.69) | -0.25 (-0.28, -0.22) | -0.21 (-0.62, 0.16) | 0.00 (-0.05, 0.03) |
| 31 | 0.12 (-0.73, 0.91) | 0.99 (0.98, 1.00) | 5.21 (4.72, 5.70) | -0.20 (-0.22, -0.18) | -0.26 (-0.66, 0.10) | 0.00 (-0.04, 0.03) |
| 32 | 0.3 (-0.18, 0.77) | 0.98 (0.97, 0.98) | 1.43 (1.21, 1.64) | -0.04 (-0.05, -0.03) | -0.24 (-0.47, -0.02) | 0.00 (-0.02, 0.02) |
| 33 | 0.98 (-0.48, 2.46) | 0.97 (0.96, 0.98) | 3.14 (2.56, 3.68) | -0.07 (-0.09, -0.05) | -0.87 (-1.56, -0.12) | 0.02 (-0.06, 0.10) |
| 34 | 0.43 (-0.22, 1.05) | 1.00 (0.99, 1.00) | 2.60 (2.36, 2.85) | -0.05 (-0.06, -0.04) | -0.45 (-0.75, -0.14) | 0.02 (-0.01, 0.05) |
| 36 | 1.38 (0.14, 2.58) | 0.97 (0.96, 0.98) | 5.65 (4.99, 6.29) | -0.23 (-0.26, -0.20) | -0.93 (-1.5, -0.36) | 0.03 (-0.02, 0.10) |
| 40A | 0.31 (-0.41, 1.06) | 0.99 (0.98, 0.99) | 2.73 (2.44, 3.01) | -0.09 (-0.1, -0.07) | -0.30 (-0.64, 0.02) | 0.00 (-0.03, 0.04) |
| 40B | 0.68 (-0.39, 1.80) | 0.98 (0.97, 0.99) | 4.45 (3.88, 5.01) | -0.12 (-0.15, -0.10) | -0.57 (-1.11, -0.04) | 0.02 (-0.03, 0.08) |
| 41 | 1.03 (-0.42, 2.41) | 0.98 (0.97, 0.99) | 2.58 (2.04, 3.19) | -0.04 (-0.06, -0.02) | -0.54 (-1.19, 0.1) | 0.00 (-0.07, 0.07) |
| 49 | -1.11 (-2.22, -0.07) | 0.99 (0.98, 1.00) | 5.67 (5.05, 6.29) | -0.15 (-0.17, -0.13) | 0.33 (-0.12, 0.78) | -0.06 (-0.11, -0.02) |
| 50 | -0.24 (-1.32, 0.88) | 0.99 (0.98, 1.00) | 0.66 (0.21, 1.09) | -0.14 (-0.16, -0.12) | -0.03 (-0.54, 0.48) | -0.03 (-0.08, 0.02) |
| 51 | -1.14 (-2.73, 0.36) | 0.98 (0.98, 0.99) | 4.08 (3.32, 4.86) | -0.13 (-0.15, -0.11) | -0.2 (-0.89, 0.49) | -0.03 (-0.11, 0.04) |
| 52 | 1.54 (-0.93, 4.29) | 0.97 (0.96, 0.98) | 4.88 (3.70, 6.05) | -0.02 (-0.04, -0.01) | -1.82 (-2.96, -0.52) | 0.10 (-0.02, 0.24) |
| 53 | -0.51 (-1.54, 0.47) | 0.99 (0.98, 1.00) | 2.62 (2.22, 3.01) | -0.15 (-0.17, -0.12) | 0.02 (-0.45, 0.48) | -0.03 (-0.08, 0.01) |
| 107 | 1.09 (-0.50, 2.74) | 0.98 (0.97, 0.99) | 4.05 (3.18, 4.83) | -0.13 (-0.17, -0.1) | -0.87 (-1.6, -0.18) | 0.04 (-0.03, 0.12) |
| 112 | 0.87 (-0.20, 2.02) | 0.98 (0.98, 0.99) | 7.10 (6.55, 7.64) | -0.11 (-0.12, -0.1) | -0.54 (-1.06, -0.04) | 0.01 (-0.04, 0.07) |
| 202 | 0.53 (-0.34, 1.38) | 0.99 (0.98, 1.00) | 3.12 (2.69, 3.53) | -0.13 (-0.15, -0.11) | -0.34 (-0.75, 0.06) | 0.00 (-0.04, 0.04) |
| 212 | -0.86 (-2.57, 0.78) | 0.96 (0.95, 0.97) | 7.86 (6.88, 8.79) | -0.22 (-0.25, -0.18) | 0.00 (-0.75, 0.72) | -0.06 (-0.14, 0.01) |
| 215 | 1.69 (0.58, 2.78) | 0.97 (0.96, 0.98) | 5.83 (5.17, 6.46) | -0.05 (-0.07, -0.02) | -0.97 (-1.46, -0.43) | 0.05 (0.00, 0.11) |

Table S2b. Median, lower, and upper 95% highest density posterior distributions for model parameters and wetland basins. Model parameters included an intercept (α), an error term (σ), precipitation (βRAIN), an autoregression term (βAR1), total precipitation over the previous seven days (βWEEKRAIN), the 12-month standardized precipitation-evapotranspiration index (βSPEI), potential evapotranspiration (βPET), and associated interactions and quadratic effects.

| ID | βWEEKRAIN | βSPEI | βSPEI:RAIN | βRAIN:AR1:SPEI | σ |
| --- | --- | --- | --- | --- | --- |
| 1 | 0.64 (0.5, 0.81) | -0.37 (-0.64, -0.09) | -0.68 (-1.21, -0.08) | 0.00 (-0.03, 0.01) | 5.36 (5.18, 5.56) |
| 2 | 0.46 (0.35, 0.57) | -0.18 (-0.38, 0.00) | 0.12 (-0.22, 0.44) | -0.01 (-0.02, 0.00) | 3.77 (3.63, 3.9) |
| 3A | 0.47 (0.34, 0.60) | -0.18 (-0.4, 0.02) | 0.47 (0.17, 0.77) | -0.02 (-0.03, 0.00) | 4.61 (4.47, 4.78) |
| 3B | 0.29 (0.20, 0.40) | -0.25 (-0.42, -0.08) | 0.34 (0.08, 0.60) | -0.02 (-0.03, 0.00) | 3.64 (3.53, 3.77) |
| 4 | 0.48 (0.33, 0.62) | 0.07 (-0.20, 0.36) | -0.57 (-0.99, -0.17) | 0.05 (0.03, 0.06) | 5.43 (5.25, 5.61) |
| 5 | 0.51 (0.33, 0.7) | 0.07 (-0.26, 0.46) | 0.11 (-0.4, 0.67) | 0.00 (-0.02, 0.00) | 6.50 (6.28, 6.74) |
| 7 | 0.33 (0.17, 0.49) | -0.15 (-0.52, 0.20) | -0.54 (-1.12, 0.00) | 0.04 (0.02, 0.05) | 5.6 (5.41, 5.81) |
| 10 | 0.52 (0.34, 0.69) | 0.24 (-0.26, 0.71) | 0.94 (-0.15, 1.98) | 0.02 (0.00, 0.05) | 4.81 (4.61, 5.07) |
| 12 | 0.28 (0.17, 0.39) | -0.14 (-0.35, 0.06) | 0.76 (0.50, 1.04) | -0.05 (-0.06, -0.04) | 3.94 (3.79, 4.07) |
| 13 | 0.47 (0.34, 0.6) | -0.06 (-0.30, 0.20) | -0.88 (-1.32, -0.43) | 0.05 (0.02, 0.07) | 4.84 (4.66, 5.01) |
| 14 | 0.29 (0.17, 0.4) | -0.13 (-0.36, 0.12) | -0.10 (-0.52, 0.29) | 0.00 (-0.01, 0.01) | 4.27 (4.1, 4.43) |
| 15 | 0.28 (0.18, 0.38) | -0.09 (-0.27, 0.07) | 0.64 (0.32, 0.92) | -0.03 (-0.05, -0.02) | 3.51 (3.39, 3.64) |
| 16 | 0.28 (0.07, 0.48) | -0.14 (-0.78, 0.46) | 3.71 (2.36, 4.97) | 0.07 (0.00, 0.14) | 4.25 (4.04, 4.47) |
| 19 | 0.26 (0.20, 0.32) | -0.02 (-0.14, 0.09) | 0.30 (0.14, 0.47) | 0.00 (-0.01, 0.00) | 2.21 (2.13, 2.29) |
| 21 | 0.14 (0.01, 0.27) | 0.20 (-0.09, 0.52) | -2.50 (-3.15, -1.83) | 0.16 (0.12, 0.2) | 4.38 (4.20, 4.57) |
| 30A | 0.25 (0.15, 0.35) | -0.03 (-0.29, 0.20) | -0.62 (-1.15, -0.13) | 0.07 (0.04, 0.11) | 3.34 (3.21, 3.47) |
| 30B | 0.29 (0.20, 0.38) | -0.16 (-0.32, 0.00) | 0.30 (-0.02, 0.64) | 0.00 (-0.02, 0.00) | 2.78 (2.68, 2.89) |
| 31 | 0.32 (0.23, 0.42) | 0.01 (-0.16, 0.19) | -0.26 (-0.83, 0.26) | 0.01 (0.00, 0.03) | 2.65 (2.54, 2.75) |
| 32 | 0.21 (0.15, 0.26) | -0.02 (-0.11, 0.06) | 0.12 (0, 0.26) | 0.00 (-0.01, 0.00) | 1.82 (1.76, 1.89) |
| 33 | 0.37 (0.21, 0.52) | -0.07 (-0.34, 0.19) | 0.51 (0.15, 0.89) | -0.02 (-0.04, -0.01) | 4.95 (4.77, 5.13) |
| 34 | 0.17 (0.11, 0.24) | 0.00 (-0.14, 0.13) | 0.83 (0.53, 1.1) | -0.02 (-0.03, -0.01) | 2.01 (1.93, 2.09) |
| 36 | 0.56 (0.42, 0.69) | -0.01 (-0.26, 0.25) | -0.86 (-1.48, -0.30) | 0.02 (0.00, 0.05) | 3.99 (3.82, 4.14) |
| 40A | 0.19 (0.12, 0.25) | -0.15 (-0.28, -0.01) | 0.82 (0.61, 1.04) | -0.03 (-0.04, -0.02) | 2.46 (2.37, 2.55) |
| 40B | 0.31 (0.2, 0.43) | -0.06 (-0.31, 0.18) | -0.48 (-1.12, 0.14) | 0.02 (0.00, 0.04) | 3.70 (3.55, 3.85) |
| 41 | 0.25 (0.13, 0.38) | 0.11 (-0.24, 0.44) | 1.21 (0.59, 1.79) | -0.04 (-0.06, -0.02) | 4.47 (4.3, 4.66) |
| 49 | 0.11 (0.01, 0.2) | 0.13 (-0.09, 0.35) | -0.36 (-0.74, 0.00) | 0.03 (0.01, 0.04) | 3.67 (3.54, 3.82) |
| 50 | 0.2 (0.10, 0.31) | 0.00 (-0.19, 0.23) | 0.63 (0.32, 0.98) | -0.02 (-0.03, -0.01) | 3.91 (3.77, 4.06) |
| 51 | 0.39 (0.25, 0.53) | 0.27 (-0.02, 0.58) | -0.25 (-0.79, 0.27) | 0.01 (0.00, 0.03) | 5.25 (5.05, 5.45) |
| 52 | 1.05 (0.79, 1.30) | -0.25 (-0.96, 0.44) | 1.45 (0.47, 2.39) | -0.02 (-0.03, -0.01) | 8.15 (7.78, 8.5) |
| 53 | 0.23 (0.13, 0.31) | 0.00 (-0.19, 0.19) | -0.03 (-0.30, 0.24) | 0.02 (0.01, 0.04) | 3.65 (3.53, 3.78) |
| 107 | 0.35 (0.21, 0.50) | -0.10 (-0.50, 0.32) | -0.79 (-1.36, -0.16) | 0.02 (0.00, 0.05) | 4.51 (4.31, 4.73) |
| 112 | 0.13 (0.03, 0.23) | -0.10 (-0.30, 0.09) | 0.65 (0.27, 1.03) | 0.00 (-0.01, 0.00) | 3.74 (3.61, 3.88) |
| 202 | 0.22 (0.14, 0.30) | -0.17 (-0.33, -0.01) | 0.59 (0.28, 0.91) | -0.03 (-0.04, -0.01) | 2.93 (2.83, 3.05) |
| 212 | 0.56 (0.40, 0.73) | 0.32 (-0.02, 0.69) | -0.68 (-1.4, 0.05) | 0.03 (0.01, 0.06) | 6.05 (5.82, 6.27) |
| 215 | 0.21 (0.08, 0.35) | -0.03 (-0.41, 0.35) | 1.41 (0.39, 2.49) | -0.05 (-0.09, -0.01) | 3.3 (3.14, 3.45) |
