# Supplementary figures and images for: Forecasting the flooding dynamics of flatwoods salamander breeding wetlands under future climate change scenarios
Source: PeerJ. 2023 Sep 19;11:e16050. doi: 10.7717/peerj.16050 (PMC10516105; doi:10.7717/peerj.16050)

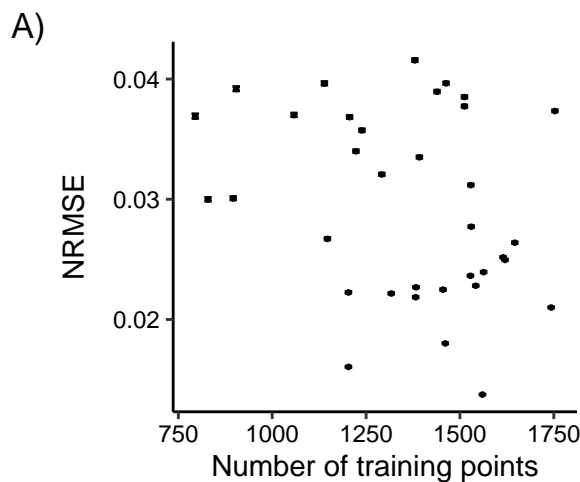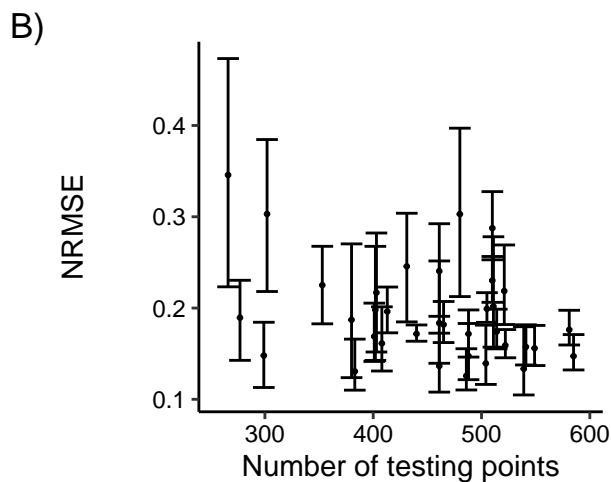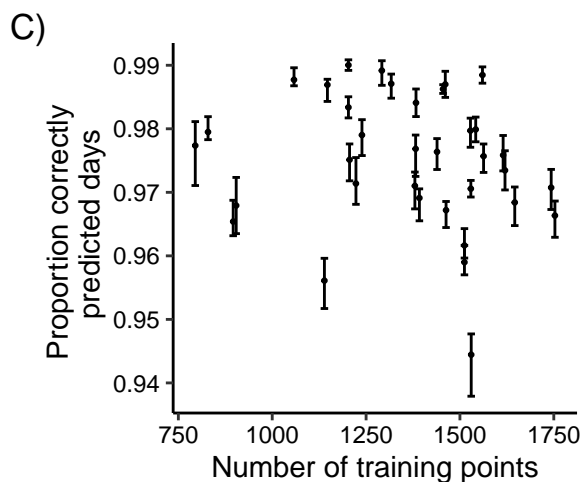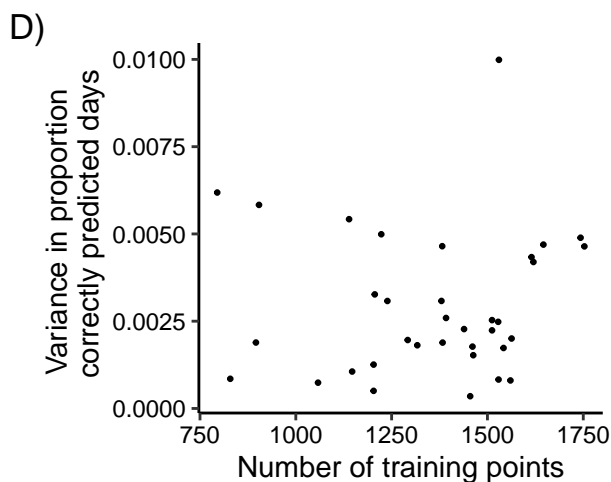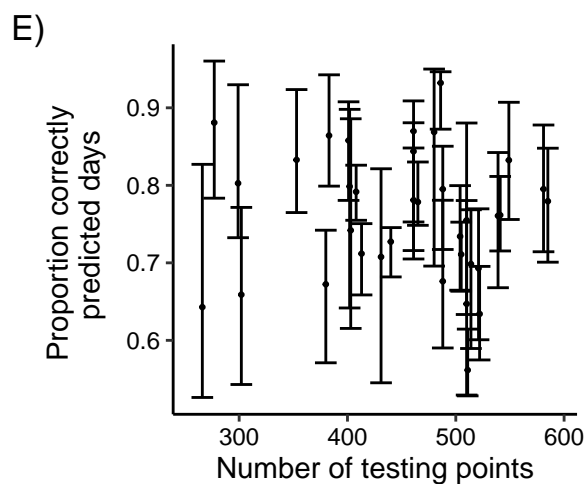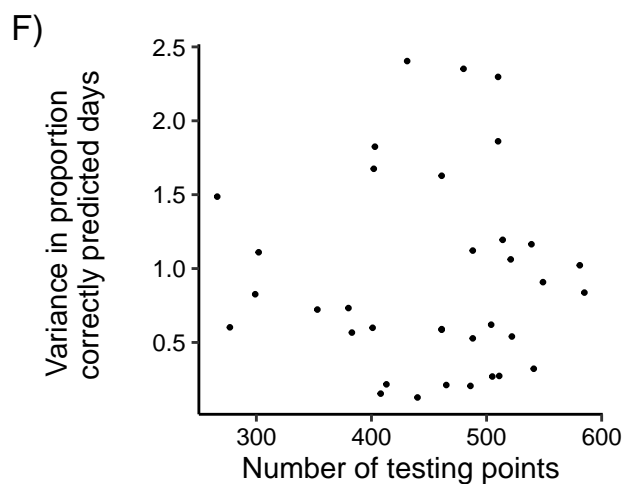

Supplement: Figure S1 — (A, B) Model accuracy for predicted water levels was estimated by normalized root mean squared error (NRMSE) and (C, D, E, F) for the prediction of length of presence of surface water by the proportion of days that correctly predicted to either have or not have water. Results are divided by the number of training (A, C, D) or testing (B, E, F) data points (daily observations) used in the model. Error bars in A, B, C, and E represent the upper and lower 95% highest density posterior and points represent median values, while points in D and F are variance estimates. Note that the y-axis scale is different for all six panels. [file peerj-11-16050-s003.pdf]

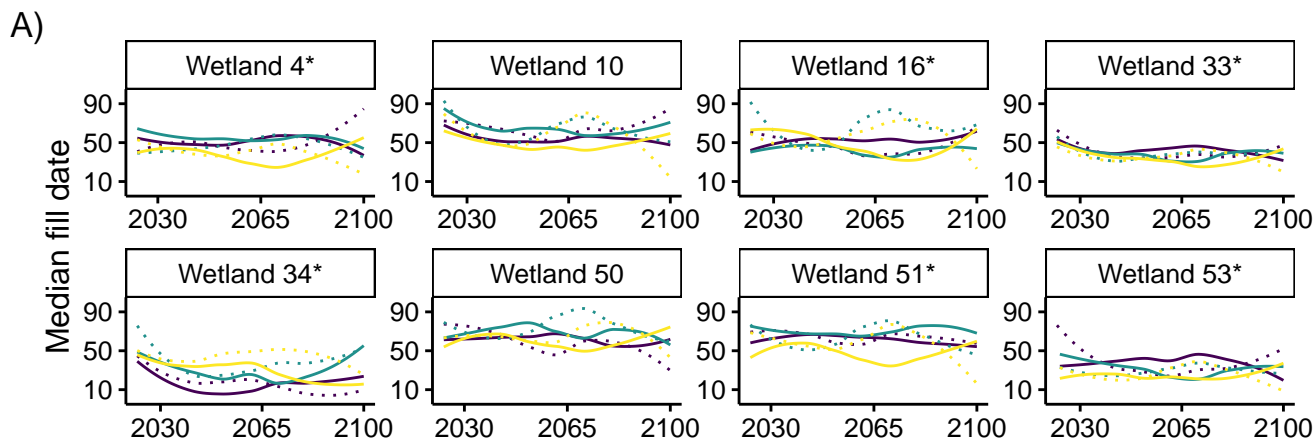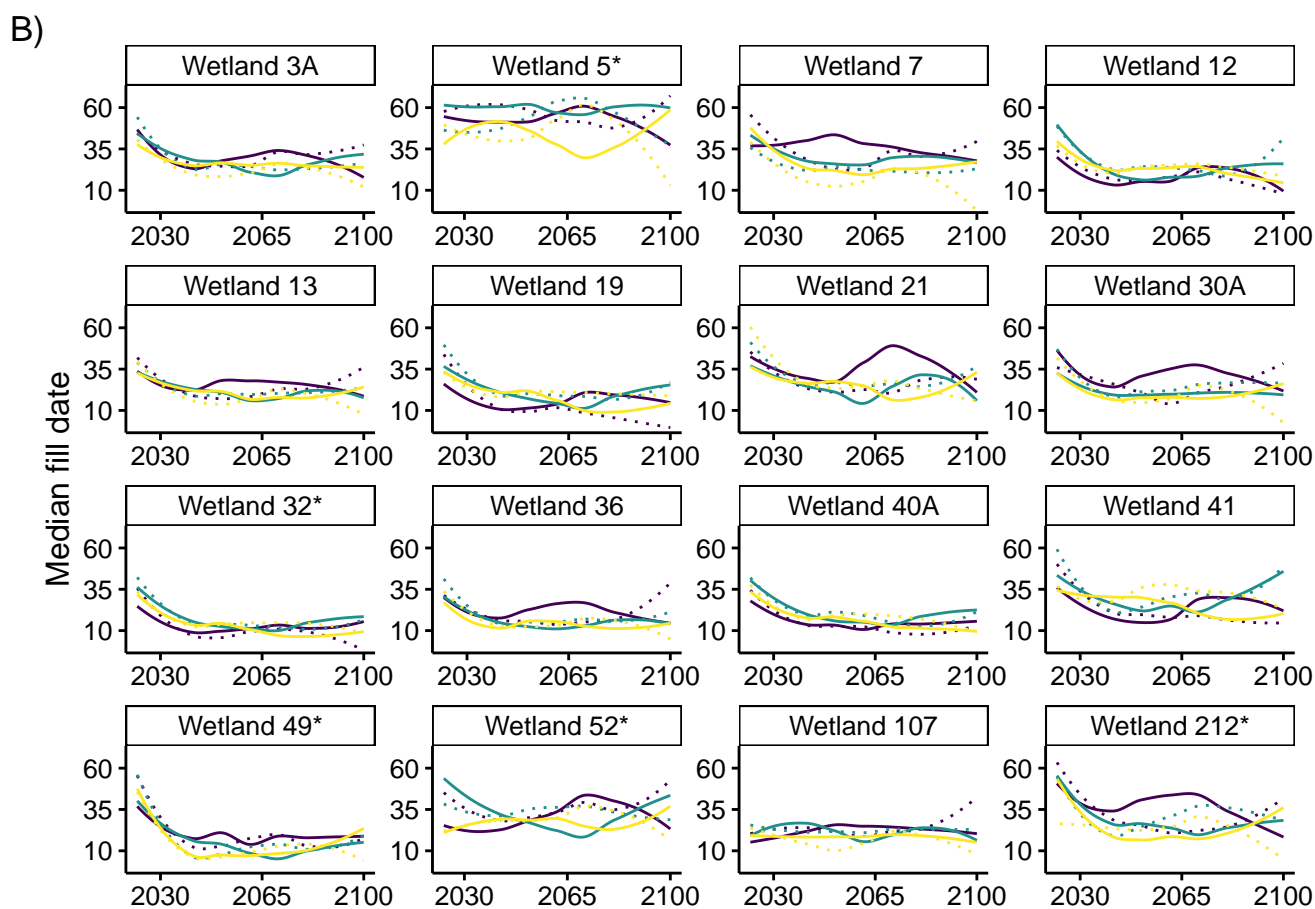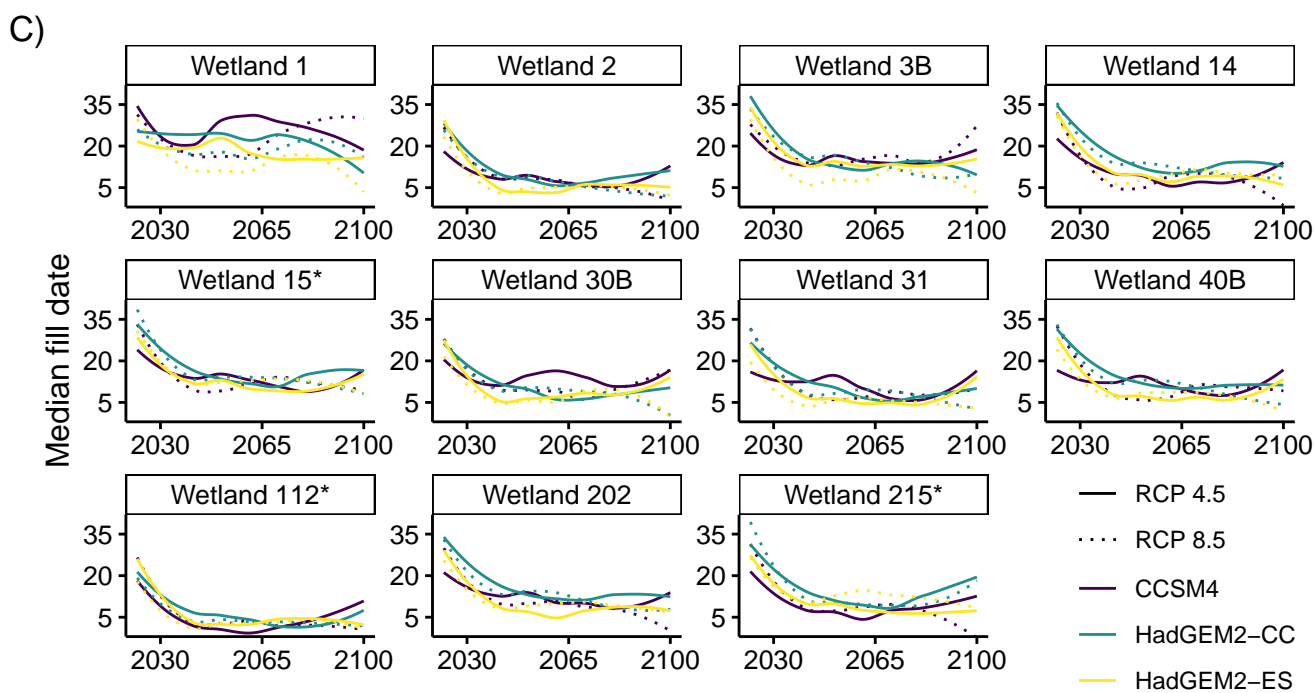

Supplement: Figure S2 — Lines represent smoothed (using the loess function in R) predictions based on 6,000 iterations of Bayesian first order autoregressive models that each simulated conditions under three global climate model (Hadley Centre Global Environment Model 2 Earth Systems [HadGEM2-ES], Hadley Centre Global Environment Model 2 Carbon Cycle [HadGEM2-CC], and the Community Climate System model version 4 [CCSM4]) and two emission scenario (RCP4.5: assumes a peak in carbon emissions in 2040; RCP8.5: assumes emissions will continue to increase throughout the 21st century) combinations (six total scenarios). Plots show overall variability in model predictions across all study wetlands. Fill dates were calculated relative to the Reticulated Flatwoods Salamander (Ambystoma bishopi) breeding season (1 November to 31 May) as days after November 1. Wetlands are grouped based on general fill date conditions: variable across approximately three months (A), two months (B), or one month (C) after November 1, and asterisks at the end of the wetland name indicate wetlands that have been confirmed occupied by flatwoods salamanders during the previous 10 years. [file peerj-11-16050-s004.pdf]
